# Establishment and characterization of a new gastric cancer cell line, XGC-1

**DOI:** 10.1186/s12935-020-01536-w

**Published:** 2020-09-05

**Authors:** Jigui Peng, Hao Xu, Jianchun Cai

**Affiliations:** 1 Department of Gastrointestinal Surgery, Zhongshan Hospital of Xiamen University, Xiamen, 361004 Fujian China; 2 Institute of Gastrointestinal Oncology, Medical College of Xiamen University, Xiamen, 361004 Fujian China; 3 Xiamen Municipal Key Laboratory of Gastrointestinal Oncology, Xiamen, 361004 Fujian China; 4 The Second Department of General Surgery, the First Hospital of Lanzhou University, No. 1, Donggang West Road, Lanzhou, 730000 Gansu China

**Keywords:** Stomach neoplasm, XGC-1, Cell line, Primary culture

## Abstract

**Background:**

To establish a primary human gastric cancer cell line.

**Methods:**

Fresh gastric cancer tissue samples were separated into a cell suspension, and DMEM/F12 medium containing 10% foetal bovine serum was used for primary culture and subculture. The morphology of the cells was observed under a light microscope, and the cell growth curve was plotted. A soft agar colony formation assay was used to detect the colony formation ability of the cell line. Immunohistochemical methods were used to detect cytokeratin, vimentin and Ki-67, the chromosome G banding method was used to analyse the karyotype of the cells, and the tumourigenic ability of the cells was detected by subcutaneous inoculation of BALB/C nude mice.

**Results:**

We established a gastric cancer cell line from a 68-year-old male patient. This gastric cancer cell line was named XGC-1 and had a doubling time of approximately 48 h. The cell line displayed strong colony formation ability and tumourigenicity in BALB/C nude mice and had complicated chromosomal abnormalities. When nutrients were insufficient, the cells shed and floated in the medium, but adherent growth was observed in nutrient-rich conditions.

**Conclusions:**

The XGC-1 cell line will be useful for future studies of gastric cancer development, progression, metastasis and therapy.

## Background

Gastric cancer is a common disease that causes digestive tract tumours and seriously threatens human life and health. Although the incidence of gastric cancer has declined in recent decades due to improvements in living conditions, the development of good eating habits, and the eradication of *Helicobacter pylori* (Hp), the incidence of gastric cancer is still high worldwide. Gastric cancer is the fourth leading cause of tumours and the third leading cause of cancer-related mortality, and its incidence in men is twice that in women [1, 2]. China has a high incidence of gastric cancer. According to the 2015 China Cancer Data Report, the estimated number of new cases of gastric cancer in China was 679,000, and the number of deaths was 498,000. The morbidity and mortality rates of gastric cancer are the second highest among malignant tumours. New cases of gastric cancer and deaths from gastric cancer in China account for 42.6% and 45.0% of the worldwide total, respectively [3].

At present, approximately 90% of gastric cancers found in China are at an advanced stage, and the prognosis of gastric cancer is closely related to the timing of diagnosis and treatment. Even if advanced gastric cancer is treated with surgery, the 5-year survival rate is still less than 30% [4]. Therefore, reducing the incidence and mortality of gastric cancer is a major public health problem that needs to be solved and will contribute to an improved quality of life for people worldwide.

The first human cancer cell line was established in a Baltimore laboratory by Gey et al. [5] over 65 years ago. Since then, many gastric cancer cell lines have been established from human tumours; these cell lines have served as important experimental tools in oncology research [6–12]. These cell lines have many advantages: they are very easy to handle, they are an infinite source of self-replicating cells, they have a relatively high degree of homogeneity, and they are easy to replace with frozen stocks if they become contaminated. However, these cell lines also have some shortcomings; cell lines easily undergo genotypic and phenotypic drift in culture, and this drift is particularly frequent in the more commonly used cell lines, especially those that have been deposited in cell banks for many years. In addition, through specific mutations, some subpopulations that show rapid growth or increased malignancy may arise over time [12–15].

A well-established tumour cell bank should reflect the diversity of tumour phenotypes and provide heterogeneous cell lines. At the same time, due to racial and regional differences, new cell lines with different backgrounds should be established, which is important for analysis of the role of regional differences in tumourigenesis and development [16]. Other important considerations for cell lines are cross-contamination, tumour heterogeneity and misidentification [17–19]. Researchers can draw erroneous conclusions or encounter serious bias when using these cell lines [14]. Despite our growing understanding of this disease, the exact molecular and genetic processes that cause gastric cancer remain largely unknown; therefore, further elucidation of the molecular pathogenesis of this disease is necessary, and the establishment of different gastric cancer cell lines will be essential.

For the above reasons, primary cell culture and the establishment of new cell lines are still important at this stage. We report a new human gastric cancer cell line, XGC-1, from a Chinese patient. This newly established cell line will be a useful model for the study of gastric cancer pathogenesis.

## Methods

### Specimen collection

The specimens used in this study were obtained with written informed consent from a 68-year-old Chinese male patient who underwent surgical resection at the First Affiliated Hospital of Xiamen University (Xiamen, P.R. China) for gastric cancer in the fundal junction of the stomach. The size of the original tumour was 3.5 × 4.3 cm. No distant metastasis was detected at the time of surgical resection. The tumour was histopathologically classified as a poorly differentiated gastric adenocarcinoma.

### BALB/C nude mice

BALB/C nude mice were purchased from Shanghai Slac Laboratory Animals Co., Ltd., and kept in the animal experiment centre of Xiamen University.

The animal experiments were carried out according to the requirements of the Xiamen University Experimental Animal Care Commission. If an animal became severely ill during the experiment, the animal was humanely killed by CO_2_ inhalation combined with cervical dislocation to confirm death and prevent animal suffering. Pathogen-free BALB/C nude mice aged 4 weeks old were housed in filter-top cages, and sterile water and food were given ad libitum. The mice weighed 15–18 g at the beginning of the experiment. All manipulations were conducted aseptically inside a laminar flow hood.

The following methods were similar or identical to those employed in previous studies [12, 20].

### Establishment of the XGC-1 cell line

Tumour specimens were rinsed twice with sterile phosphate-buffered saline (PBS) containing antibiotics. Then, the samples were enzymatically disaggregated after incubation with collagenase type II and neutral protease solution at 37 °C in a humidified atmosphere containing 5% CO_2_. After the samples were incubated for approximately 0.5 h, 5 ml of foetal calf serum (FBS) (Gibco, Grand Island, NY, USA) was added to terminate the digestion. Then, the digested tumour fragments and fluid were filtered through a 200 mesh sieve, and the filtrate was centrifuged at 1000 rpm for 5 min. The supernatant was removed, and the remaining cells were resuspended in Dulbecco’s modified Eagle’s medium (DMEM) and Ham’s F-12 medium (1:1) (Gibco) supplemented with penicillin (100 U/ml), streptomycin (100 μg/ml), heat-inactivated 2% FBS, hEGF (0.1 ng/ml), bFGF (0.1 ng/ml), hydrocortisone (25 μg/ml), fluconazole (40 μg/ml) (Gibco), and M-plasmocin (25 μg/ml) (Invitrogen); seeded in 12-well culture plates; and cultivated at 37 °C in a humidified atmosphere of 5% CO_2_ in air. The growth medium was replaced every 2–3 days, and the plate was regularly assessed for epithelial cell and fibroblast outgrowth. When the cells completely covered the bottom of the plate, they were passaged. Currently, the cell line has been cultured for > 90 passages. The cells were tested for mycoplasma contamination, and the result was negative. The cell line was designated XGC-1 (Xiamen gastric cancer-1).

### Short tandem repeat analysis for authentication

To authenticate the established cell line, we analyzed short tandem repeats (STRs) of the XGC-1 cells and the original tumor tissue. The data were analyzed, and the STR profiles were compared with those recorded in public cell banks, such as the ATCC, CCTCC, and JRCB, for reference matching.

### Morphology of the XGC-1 cell line

XGC-1 cells were seeded in 6-well plates and incubated at 37 °C in a humidified atmosphere containing 5% CO_2_ for 2 weeks. Every day, the cells were placed under an inverted microscope to observe their general morphology. Cell ultrastructure was observed under a transmission electron microscope.

### Cell growth properties

Cells were plated in 96-well plates at 1000 cells/well and cultured in DMEM/F12 containing 10% FBS for various durations. Cell numbers were measured by MTT assays, which were performed according to the manufacturer’s protocol. The doubling times were determined from the growth curve.

### Colony-forming efficiency

Colony formation of different cell clones in soft agar was used to assess their growth capabilities. A single-cell suspension (1 × 10^4^ cells/ml) was prepared. Agar (0.6%) was mixed with DMEM/F12 and added to 6-well plates as the lower layer. Then, 0.3% agar was mixed with the cell suspension, and the mixture was added to the same 6-well plate as the upper layer. Every well contained 1000 cells. The plates were incubated at 37 °C in a humidified incubator containing 5% CO_2_. Fourteen days later, cell colonies were counted with a microscope, and the colony formation rates were calculated using the following formula: colony formation rate (%) = (number of colonies/number of cells inoculated) × 100%.

### Chromosome analysis

Cells were karyotyped using a standard air-drying method after treatment with a final concentration of 0.05 mg/ml colcemid for 2 h when the cells were in the exponential growth phase. The cells were analysed using trypsin G banding. A total of 200 metaphase spreads were examined to determine the modal number. Karyotyping was performed according to the International System for Human Cytogenetic Nomenclature (2005). Chromosome analysis was carried out on the cell line at passage 40.

### Tumourigenicity in BALB/C nude mice

The study protocol for mice was approved by the Xiamen University Experimental Animal Care Commission. Briefly, cells at passage 45 were prepared to determine their tumourigenicity in BALB/C nude mice. Cultured cells (1 × 10^6^ cells/ml) were harvested, washed, resuspended in 0.1 ml of complete DMEM/F12, and injected subcutaneously into the right flanks of three 4-week-old female BALB/C nude mice. The animals were examined every week for the development of tumours. Tumour-bearing mice were sacrificed. Tumour tissue was excised, fixed in 10% formalin, and processed for routine histopathological examination.

### Immunohistochemistry

Cell monolayers at passage 35 were subcultured and grown on sterile microscope slides. After confluent growth was observed, the slides were washed with PBS, fixed in 4% paraformaldehyde for 15 min, air-dried and treated with 0.5% Triton X-100 for 20 min. PBS was used as the negative control, and the slides were then overlaid with the following antibodies: a mouse monoclonal antibody directed against human cytokeratin, a rabbit monoclonal antibody directed against Ki-67, and a mouse monoclonal antibody directed against human vimentin, mouse monoclonal antibody directed against human carcinoembryonic antigen, mouse monoclonal antibody directed against human cancer antigen 19-9. The slides were incubated with the antibodies for 60 min and thoroughly washed with PBS. Biotinylated rabbit anti-mouse IgG was subsequently applied for 15–20 min, and the samples were washed. Then, a solution of DAB was added to the slides, and the slides were incubated for 1–5 min at room temperature. Finally, the slides were rinsed with distilled water, counterstained with haematoxylin and eosin (H&E) and examined by light microscopy.

### Statistical analysis

The statistical significance of differences between experimental groups and controls was determined by Student’s t-test. Values of P ≤ 0.05 were considered statistically significant.

## Results

We successfully established a new gastric cancer cell line, XGC-1, in vitro using a fresh sterile specimen derived from the primary tumour of a gastric cancer patient. It was confirmed that XGC-1 cells did not correspond to any cells deposited in public cell banks (Table 1). From the primary culture, we succeeded in freezing, thawing and subculturing cells for > 90 generations in DMEM/F12 supplemented with 10% FBS.

**Table 1:** Results of STR analysis

Microsatellite (chromosome)	XGC-1 (P90)	Tumor tissue
Amelogenin	X, Y	X, Y
TH01	9	9
D21S11	29	29
D5S818	10,13	10,13
D13S317	9,11	9,11
D7S820	11	11
D16S539	11,12	11,12
CSF1PO	11,13	11,13
vWA	16,17	16,17
TPOX	8	8

P indicates the passage number of a cell line

### In vitro *characteristics of XGC*-*1 cells*

**Fig. 1:**
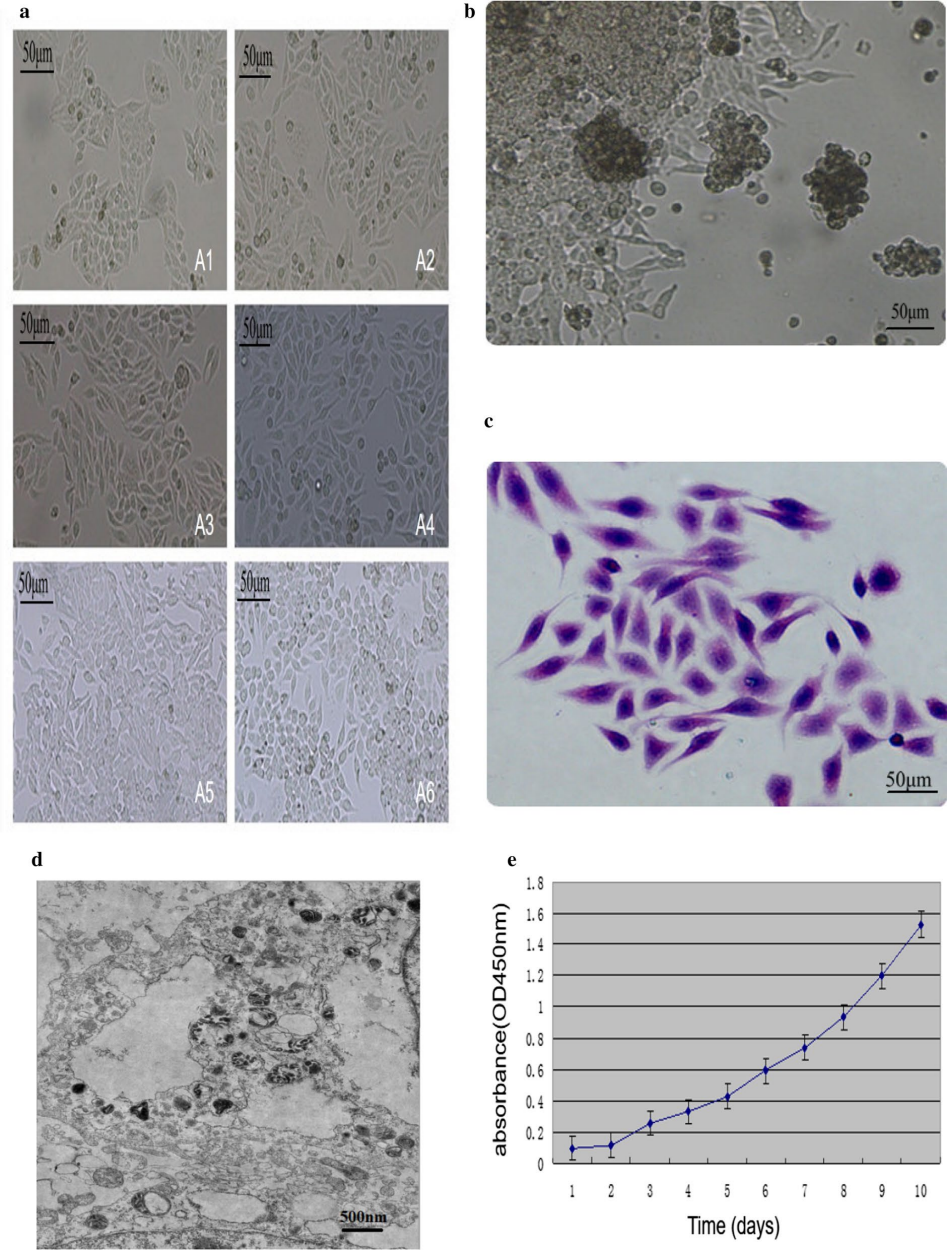
Morphology of XGC-1 cells. **a** Micrographs of XGC-1 cells at different passages under phase contrast (A1: passage 1; A2: passage 10; A3: passage 20; A4: passage 30; A5: passage 40; A6: passage 50. Magnification, ×200). **b** When nutrients are insufficient, the cells will fall off the plate into the culture medium (magnification, ×200). **c** H&E staining showed large nuclei and prominent nucleoli (magnification, ×200). **d** Ultrastructural appearance of XGC-1 cells (magnification, ×8000). **e** The growth curve of XGC-1 cells

Under light microscopy, most of the cells appeared spindle-shaped and oval-shaped, a few cells were polygonal, the cell poles appeared sharp, the nuclei were obvious, a few cells were multinucleated, and the nucleolus was clearly visible (Fig. 1a). The cells aggregated and grew during culture. When the nutrient level was insufficient, the cells floated in the culture medium, and they continued to adhere to the wall in nutrient-rich conditions (Fig. 1b). H&E staining showed that the nuclei were deeply stained and the proportion of nucleoplasm was high, which is consistent with cancer cell characteristics (Fig. 1c). Transmission electron microscopy revealed that most cell nuclei increased in volume, were round or elliptical, and had a large proportion of nucleoplasm. Chromatin in the nucleus was often significant, part of the nuclear membrane was recessed, and the nucleolus was significantly enlarged and solid; the number of free ribosomes increased and there were fewer organelles, mitochondrial swelling was obvious, the crest morphology was irregular and the crest number was small, the connection between the cells was scarce, and there were no microvilli structures (Fig. 1d). The cells were passaged > 90 generations, and there was no obvious change in morphology during cell passage. The growth of the XGC-1 cell line was assayed by the MTT method. The XGC-1 cell line showed vigorous growth and a doubling time of approximately 48 h (Fig. 1e).

### Colony-forming efficiency

**Fig. 2:**
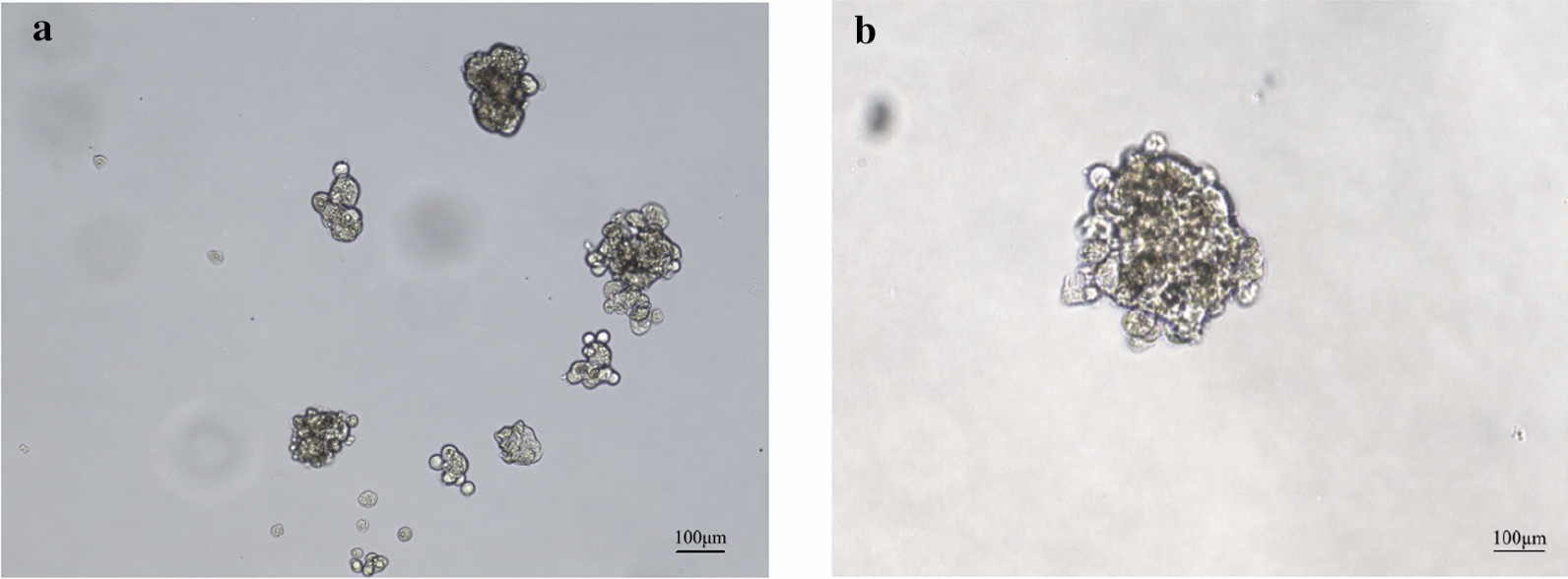
The anchorage-independent growth capability of XGC-1 cells in vitro. XGC-1 cells were plated in 0.3% agar supplemented with 10% FBS for 2 weeks to test the formation of colonies. **a** Colony formation for 1 week. **b** Colony formation for 2 weeks

The cells began to divide on the third day of culture in low-melting agarose, and colonies were formed in the independent single-cell culture at 2 weeks. The cells were stacked in a circular shape, and white colonies were observed with the naked eye at 3 weeks. The colony formation rate was approximately 72.0% (Fig. 2a, b).

### Karyotype of the XGC-1 cell line

**Fig. 3:**
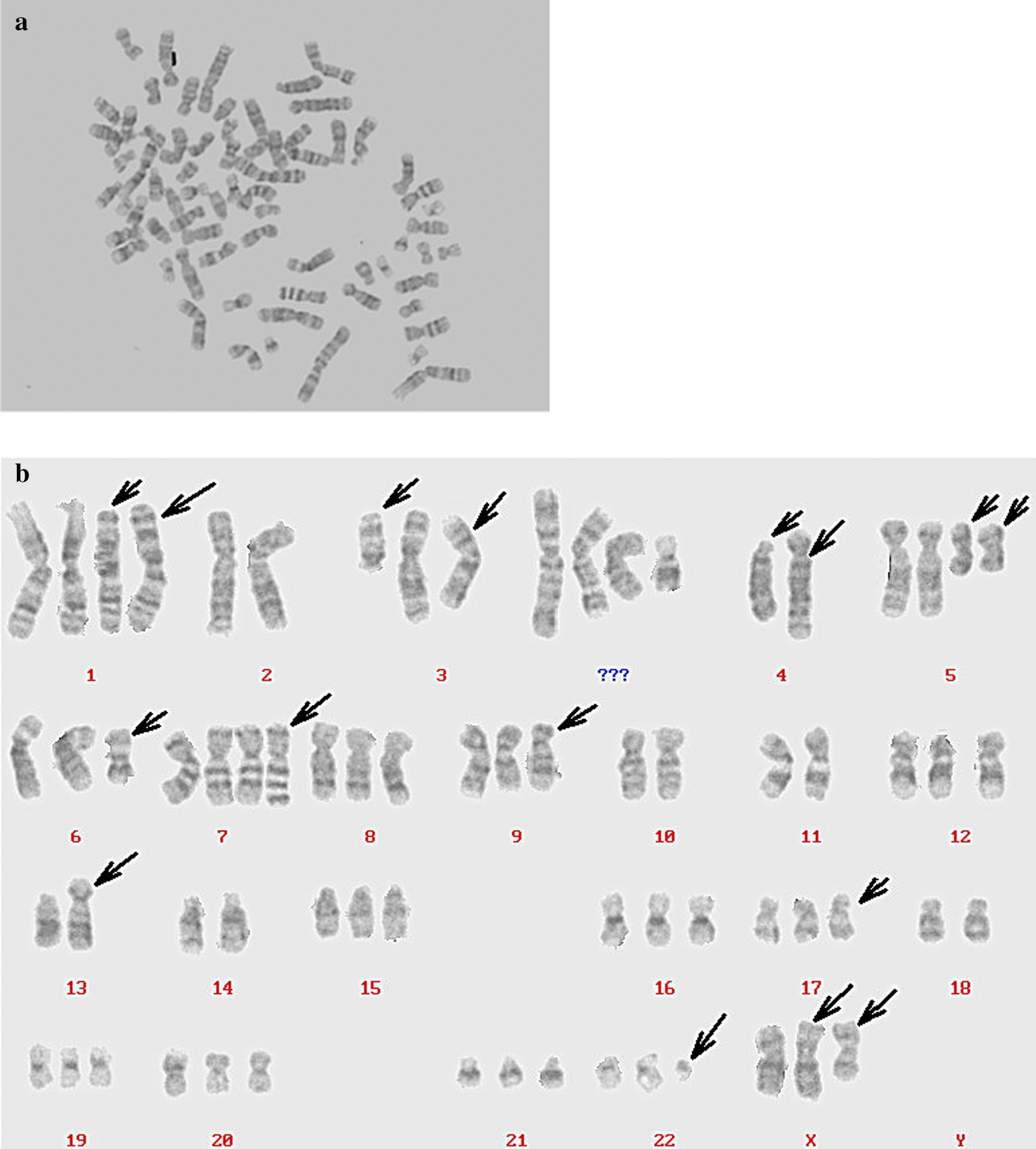
Karyotype analysis of the XGC-1 cell line. The karyotype of the XGC-1 cell line shows abnormalities in both the number and structure of chromosomes

The karyotype was triploid, many deletions were observed, ectopic and derived chromosomes (→) were visible, the X chromosome had an abnormal arm, the Y chromosome was lost, no normal chromosome 3 was present, the Philadelphia chromosome (Ph’) was observed, and 4 chromosomes with unknown sources were detected. The representative karyotype was 69, X, −Y, der(X)add(X)(P?), i(X)(p10), der(1)add(1)(p?), der(1)add(1)(p?), −2, del(3)(q10), der(3)t(3;5) (p10;q10), der(3)t(3;9)(q10;p10), −4, del(4)(p10), der(4)del(4)(p15)add(4)(q?), −5, i(5)(p10) × 2, del(6)(q21), +der(7)add(7)(p?), del(9)(p21), −10, −11, −13, der(13)add(13)(p?), −14, del(17)(q24), −18, del(22)(q11), +mar1, +mar2, +mar3, +mar4 (Fig. 3a, b).

### Histological examination

**Fig. 4:**
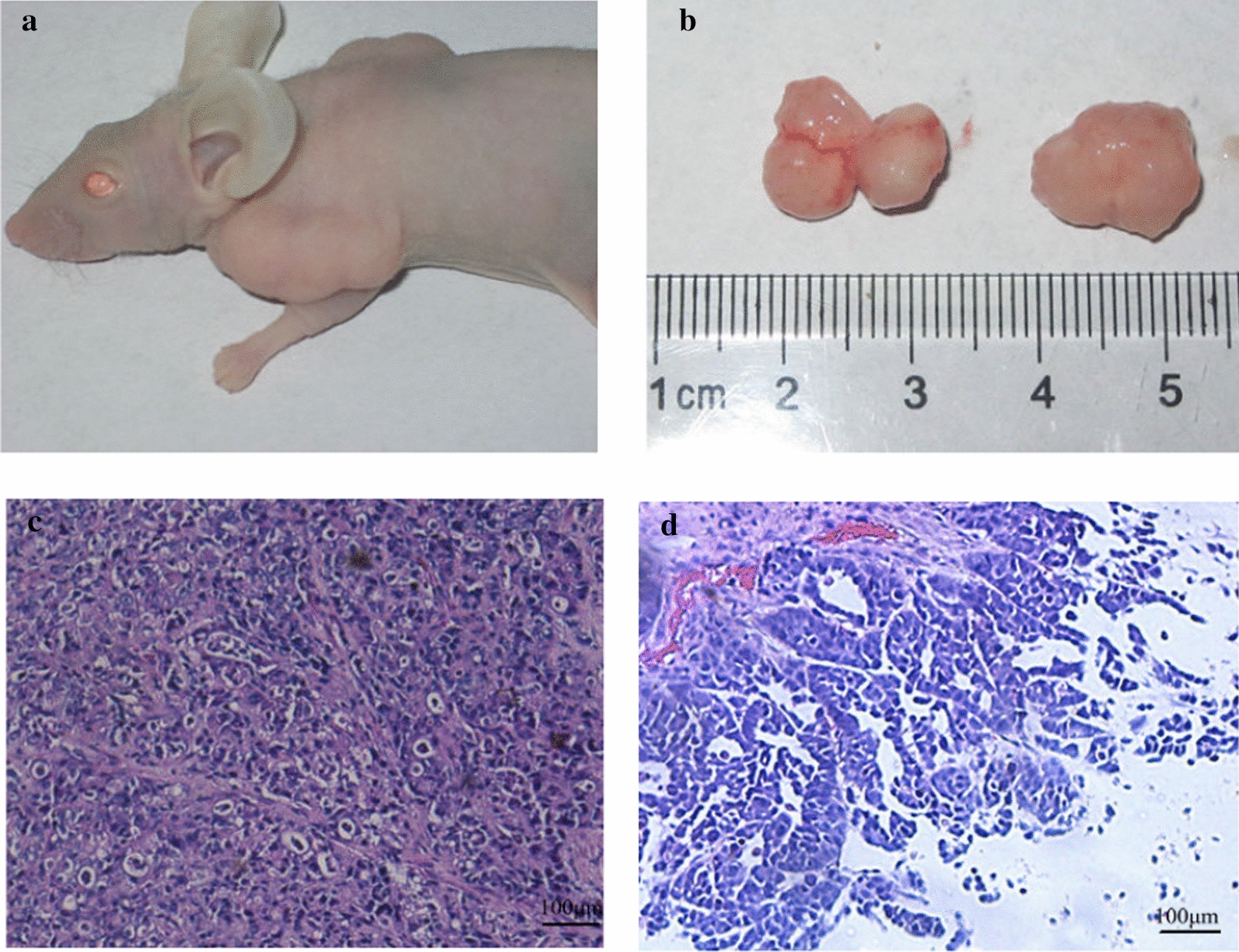
Tumourigenicity of XGC-1 cells in nude mice. **a** BALB/C nude mice were injected subcutaneously with 1 × 10^6^ cells for 4 weeks, the mice were sacrificed, and the tumours were excised for histopathological examination. **b** The solid tumour was obtained. **c** Histology of primary tumours formed by XGC-1 cells showed poorly differentiated gastric cancer, and the structure of the gland was seen (magnification, ×100) (H&E). **d** Histology of xenografted tumours of XGC-1 cells showed poorly differentiated gastric cancer, and the structure of the gland was observed (magnification, ×100) (H&E)

Subcutaneous inoculation for 5 days resulted in a mung bean-sized tumour. One month later, the BALB/C nude mice were sacrificed (Fig. 4a). The subcutaneous tumours were removed for histological examination (Fig. 4b) and were compared with the original primary tumour. Both primary tumours and subcutaneous tumours (Fig. 4c, d) were characterized by typical gastric adenocarcinoma (H&E) features, and all were poorly differentiated. The liver, kidney, lung and other important organs showed no tumour metastasis.

### Immunohistochemistry

**Fig. 5:**
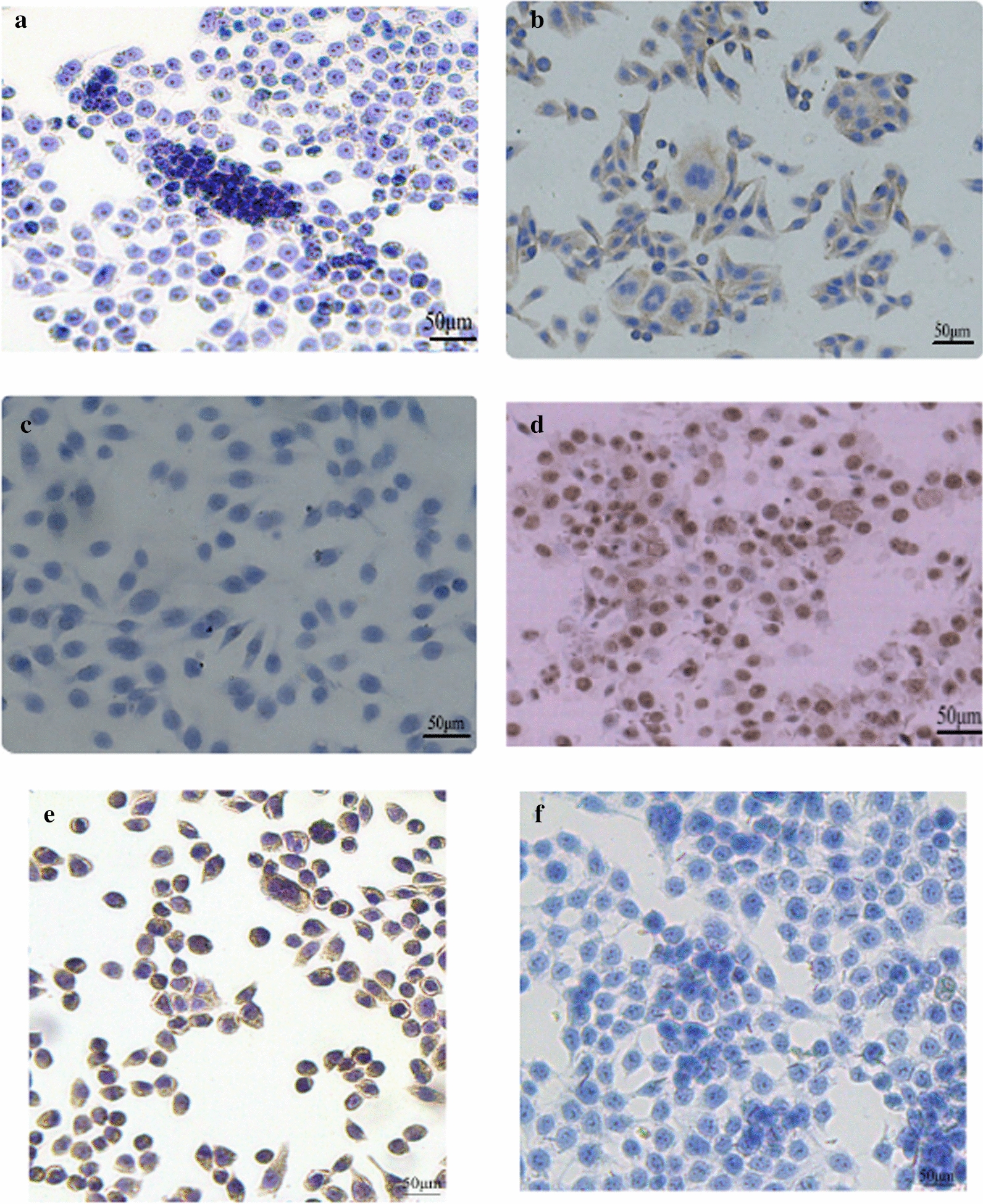
Immunohistochemistry of XGC-1 cells (magnification, ×200). **a** PBS was used as a negative control. **b** XGC-1 cells exhibited positive results for cytokeratin. **c** The cells were negative for vimentin. **d** Ki-67 was expressed at very high levels in the nucleus (≥ 75%). **e** Carcinoembryonic antigen was positive in XGC-1 cells. **f** Cancer antigen 19-9 was negtive

PBS was used as the negative control (Fig. 5a). The results of immunohistochemical staining revealed that cytokeratin expression was positive (Fig. 5b), and cytoplasmic keratin was stained a tan colour. The membrane and nucleus were not stained, and vimentin expression was negative (Fig. 5c). Cytoplasmic vimentin staining was not observed, which was consistent with the epithelial source characteristics. Nuclear Ki-67 was stained brown (Fig. 5d). Carcinoembryonic antigen was positive in XGC-1 cells (Fig. 5e), while cancer antigen 19-9 was negtive. The patina and capsule were not stained, and 5 fields were randomly observed under a microscope. The average staining rate of the fields of view was approximately 75%.

## Discussion

The establishment of various gastric cancer cell lines can provide materials for analysing the characteristics of different types of gastric cancer. By analysing the characteristics and commonalities of different gastric cancer cell lines, researchers can reveal the mechanisms underlying the occurrence and development of gastric cancer and lay a theoretical foundation for the clinical treatment of gastric cancer. Recent studies have also found that differences in regional factors can affect tumour characteristics in cancer patients, leading to different clinical outcomes [16]. For the above reasons, primary cell culture and the establishment of new cell lines are still important at this stage.

In this study, a gastric cancer cell line (XGC-1) derived from a primary tumour that was shown to have characteristics of poorly differentiated adenocarcinoma was established from a male Chinese patient. Immunohistochemistry of the gastric cancer cell line showed that the cells were derived from epithelial cells, cell proliferation was active, and staining for the proliferating cell nuclear antigen Ki-67 was ≥ 75%. Ki-67 is a cell cycle-associated protein that is closely related to tumourigenesis and development. The degree of Ki-67 expression can reflect the proliferation of tumours and can be used to predict the prognosis of patients; patients with high expression have a poor prognosis [21–23].

In this cell line, single cells cultured in agarose can grow independently and proliferate, and the doubling time does not change significantly. The colony formation rate is high, clones can be formed in 2 weeks, and cell colony formation can be observed with the naked eye at 3 weeks. At the same time, the tumour formation experiment in nude mice confirmed that 100,000 cancer cells can form tumours under the skin, and the growth rate of the tumour is rapid. The tumour size of a grain of rice can be observed within 5 days of inoculation, and the volume at 1 month reached 1 × 1.5 cm^3^. These characteristics indicate that the cell line has high cell viability, inactivation of the growth regulatory mechanism, strong cell independence, and strong metabolism. Thus, this line can be used for analysis of cancer stem cells and the mechanisms regulating cell proliferation and can also be used for related research on treatments and tumour apoptosis. Infinite proliferation is one of the characteristics of tumour cells. This cell line has been passaged for > 90 generations. During the culture process, the cells can be stably passaged, and the morphology is unchanged.

For a human-derived malignant tumor, it is generally considered that it has strong invasion and metastasis capabilities, but for nude mice, this malignant tumor is actually just a benign tumor, and the differences between species also affect tumor cells. For example, if a low-grade human-derived tumor cell is injected into the tail vein, it may not grow in nude mice, and a highly malignant tumor cell is rarely seen in nude mice. Especially for studies focusing on tumor metastasis, the nude mouse experimental model will cause great difficulties in the interpretation of subsequent results. Because the metastatic behavior is closely related to the environment, nude mice have no T cell immunity and a xenogenic growth environment. Of course, the site of tumor cell inoculation may also be related to the occurrence of metastasis. The influence of these factors on tumor metastasis cannot be ignored, and the conclusion of the experiment should be cautious.

The cells can be superimposed on each other and grow in a mass. When the nutrient level is insufficient, the cells will fall off the surface and float, and when the nutrient level is sufficient, they will reattach to the wall. Although the established cell line was derived from in situ gastric cancer tissue, its growth pattern indicates that the cell line has metastatic tumour characteristics, which may be related to high malignancy and poor prognosis. Whether the cell adhesion-shedding-adherent growth progression is the same as the mechanism of clinical tumour metastasis and whether the overgrowth of tumour cells leads to a lack of cell nutrition and tumour metastasis remain unknown. The relevant effects on prognosis should be examined in the future.

Chromosomal instability is mainly manifested by changes in chromosome structure and quantity. The chromosomes of early tumour cells are mostly diploid, and chromosome instability increases with tumour progression and is often accompanied by changes in chromosome number and the generation of hybrid DNA [24, 25]. In gastric cancer, chromosomal instability is one of the main causes of genetic instability. Chromosomal deletion, cleavage and rearrangement lead to the activation of oncogenes or the inactivation of tumour suppressor genes, which promotes tumourigenesis and development [26, 27]. Therefore, the establishment of a gastric cancer cell line with chromosomal instability is conducive to study the relationship between the occurrence of gastric cancer and genetic instability. In this gastric cell line, many chromosomal deletions and ectopic and derived chromosomes were found. Among them, the Y chromosome was lost, chromosome 3 was obviously changed, and no normal chromosome 3 was observed. The karyotype also showed the Philadelphia chromosome and chromosomes with an unknown source. Abnormal chromosome 3 is present in many gastric cancer cells and is closely related to the occurrence of gastric cancer. Abnormal chromosome 3 causes the activation of oncogenes or the inactivation of tumour suppressor genes, leading to the occurrence of gastric cancer [28]. Previous studies found that the common fragile site on chromosome 3 is unstable during DNA replication and is prone to heterozygous ectopic loss [29]. Ectopic loss of this site results in gene inactivation of a tumour suppressor gene, fragile histidine trimer (FHIT). Low expression of the FHIT gene plays an important role in the prognosis of gastrointestinal tumours [30]. The gastric cancer cell line established in this study may be used to study the relationship between gastric cancer and chromosome 3.

## Conclusions

In conclusion, we report the establishment and characterization of a new human gastric cancer cell line that was derived from a primary tumour and named XGC-1. The gastric cancer cell line has strong cell viability, a high tumour formation rate, high malignancy and metastatic ability. This line can be used as a resource for analyses of the mechanisms underlying the pathogenesis, invasion and metastasis of gastric cancer and as a foundation for diagnosis and treatment.

## Data Availability

The datasets used and/or analysed during the current study are available from the corresponding author upon reasonable request.

## Abbreviations

Hp: *Helicobacter pylori*
PBS: Phosphate-buffered saline
FBS: Foetal bovine serum
DMEM: Dulbecco’s modified Eagle’s medium
H&E: Haematoxylin and eosin
Ph’: Philadelphia chromosome
FHIT: Fragile histidine triad
